# The mediating roles of activities of daily living and depression in the relationship between pain and sleep duration among rural older adults in China: a cross-sectional study

**DOI:** 10.3389/fpubh.2025.1543474

**Published:** 2025-03-28

**Authors:** Yanxu Liu, Guoqi Fu, Yulin Chai, Cailing Xue, Qi Song, Sheng Luo, Li Luo

**Affiliations:** ^1^School of Management, Shandong Second Medical University, Weifang, China; ^2^Medical Insurance Office of Weifang People’s Hospital, Weifang, China

**Keywords:** pain, activities of daily living, depression, sleep duration, rural older adults

## Abstract

**Background:**

As the population ages, the health of rural older adults is of increasing concern to society. Pain, decreased activity of daily living, depression, and sleep are important factors affecting the quality of life of older adults. This study aimed to explore the complex relationship between pain, activity of daily living, depression, and sleep in rural older adults, with the goal of providing new perspectives and intervention strategies to improve sleep quality.

**Methods:**

This study was based on the data from the 2020 China Health and Retirement Longitudinal Study, and rural older adults aged 60 years and above were selected as the study subjects, with a final sample size of 5,352. Stata 18.0 and SPSS 27.0 software were used for statistical analysis, and *t*-tests, analysis of variance (ANOVA), and Pearson correlation analyses were used for one-way analyses, and PROCESS 4.2 was used for mediation effect analysis and testing.

**Results:**

Pain in older adults was negatively correlated with sleep duration (*r* = −0.212) and positively correlated (*p* < 0.001) with impairment to activity of daily living (*r* = 0.339) and depression (*r* = 0.355). The mediation test reported that pain in older adults had a direct effect on sleep duration (95% CI: −0.076 to −0.043), with activity of daily living (95% CI: −0.014 to −0.004) and depression (95% CI: −0.026 to −0.017) acting as chained mediators between the two.

**Conclusion:**

This study reveals the interrelationships between pain, activity of daily living, depression and sleep in rural older adults. It is recommended that medical resources be strengthened, health awareness be increased, community care services be improved, recreational activities be provided, and family emotional support be encouraged to improve the health and quality of life of older adults.

## 1 Introduction

According to data from the seventh China Population Census in 2020, the number of older adults aged 60 years and over in China had reached 264 million, accounting for 18.73% of the total population (1). With the acceleration of the aging process, especially in rural areas, the proportion of people over 60 years of age in the total rural population is 23.81 per cent, which is 7.99 percentage points higher than that in towns and cities (2). Compared with urban areas (3), the lower level of economic development in rural areas, the scarcity of medical resources, the lagging infrastructure, the insufficient insurance coverage and the high rate of traffic accidents make the health condition of the majority of rural older adults not objective (4, 5).

Pain is a common health problem in the older population, with more than two-thirds of older adults suffering from pain (6), which is costly (7), often accompanied by sleep problems, such as shortened sleep duration or decreased sleep quality (8), and can easily lead to insomnia (9). Studies have shown that there is a strong relationship between pain and sleep problems, with pain sufferers having a 70% or higher probability of sleep problems compared to those without pain (10). Chronic pain not only leads to sleep deprivation, but also triggers a range of physical and psychological complications (11).

Sleep is an important anabolic process for cell and tissue regeneration, and humans spend about one-third of their time sleeping, and good sleep is essential for good health (12, 13). However, pain is not the only factor affecting sleep, as studies have shown (14–17) that activity of daily living (ADL), an important measure of physical activity, and depression, an important indicator of mental health, play an important role in this process and are both considered risk factors for sleep. In addition, pain often leads to limited mobility, interfering with daily activities and triggering elevated negative emotions, which can affect independence and quality of life (15). Impaired activity of daily living can further affect sleep (18). In turn, older adults with lower mental health tend to experience more severe sleep problems due to the combined stress of their emotional, psychological and physical state (19).

In summary, although scholars at home and abroad have explored research on pain, activity of daily living, depression, and sleep, most of them have focused on only two or three factors. There is a lack of systematic research on links among all four factors. In addition, most of the existing studies have focused on the older adult population, and there is still a paucity of relevant research on the Chinese rural older adult population. Therefore, the present study focused on rural Chinese older adults, aiming to explore the complex relationship between pain, activity of daily living, depression and sleep duration, and to provide new perspectives and intervention strategies to improve the sleep of rural older adults, and to promote their physical and mental health and quality of life.

## 2 Methods

### 2.1 Data sources

**Figure 1:**
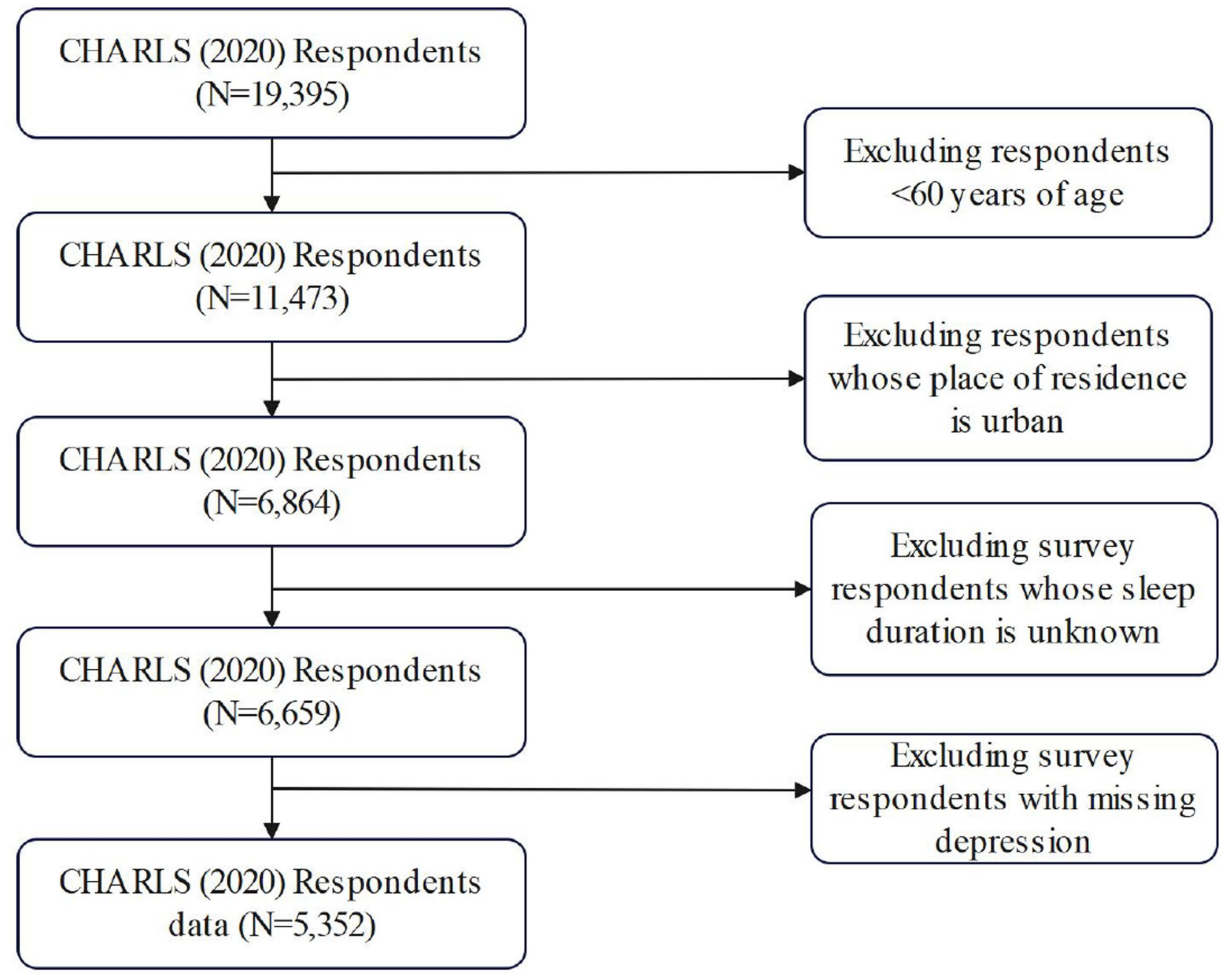
Flow chart of sample inclusion.

The data base for this study come from the 2020 China Health and Retirement Longitudinal Study (CHARLS), which utilized implicitly stratified PPS random sampling for the baseline survey. 19,395 people were sampled in the 2020 CHARLS. The CHARLS survey was approved by the Biomedical Ethics Committee of Peking University (IRB00001052-11015). In this study, rural older adults over 60 years old were selected as the study population, and 5,352 samples were finally obtained. The specific screening process is shown in Figure 1.

### 2.2 Measures

#### 2.2.1 Pain

**Figure 2:**
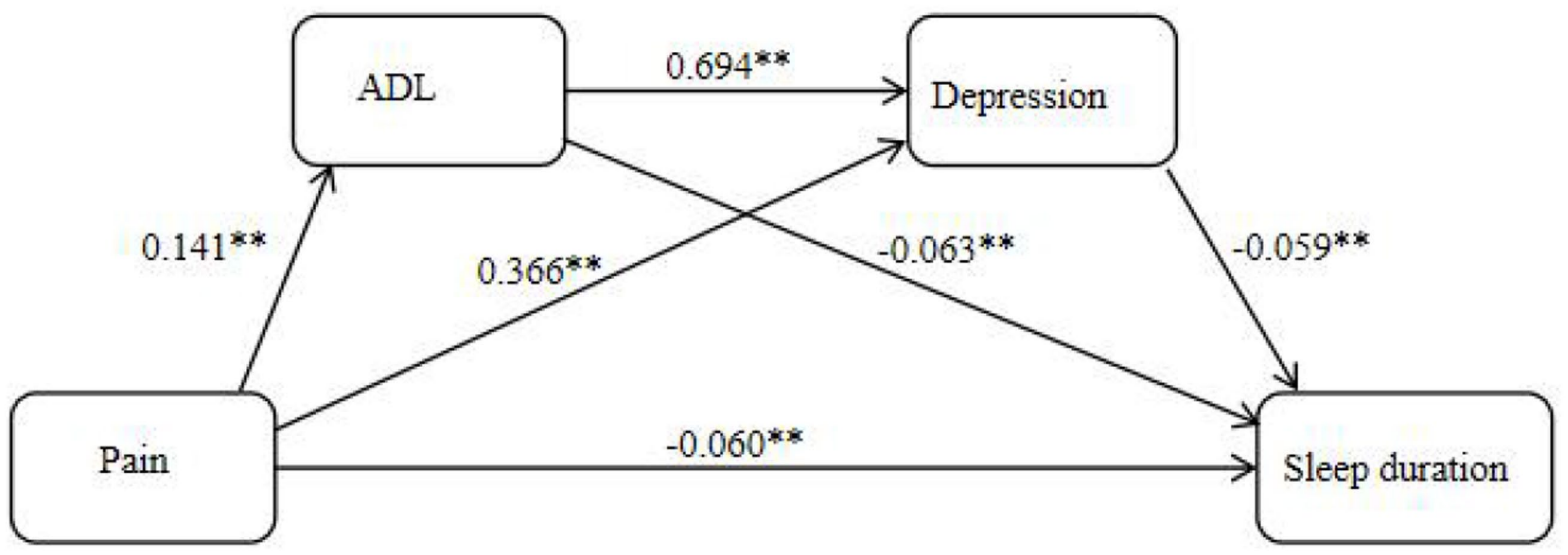
A chain-mediated model of pain on sleep duration. **p < 0.01.

The core independent variable of this study was pain (20). CHARLS asked respondents about 15 possible pain sites (including head, shoulder, arm, wrist, finger, chest, stomach, back, lumbar, hip, leg, knee, ankle, toe, and neck). Subjects were scored 1 point for each site of pain they reported, with a total score ranging from 0 to 15. Higher scores indicate more pain for the respondent. In this study, the scale Cronbach alpha coefficient was 0.898 (Figure 2).

#### 2.2.2 Sleep duration

The dependent variable in this study was sleep duration, a measure of nighttime sleep time that has been widely used in Chinese studies (21). The CHARLS determines sleep duration by asking respondents, “In the past month, on average, about how many hours did you actually fall asleep each night.”

#### 2.2.3 Activities of daily living and depression

The mediating variables in this study included activity of daily living and depression. These scales have been widely used in domestic and international studies and have been verified to have good reliability and validity (22–24). Activity of daily living (ADL) in CHARLS was measured by using the Katz scale, which consists of six items, including bathing, dressing, toileting, getting in and out of bed, eating, and controlling urination and defecation. Subjects self-assessed each activity according to their own situation and were categorized into four levels: “no difficulty,” “difficulty but can still do it independently,” “difficulty and need assistance,” and “unable to do it at all.” The total score ranged from 0 to 18, with higher scores indicating more severe impairment in the activity of daily living. For depression, the short version of the Center for Epidemiological Studies Depression Scale (CES-D10) was used. The scale contains 10 entries, each of which is categorized into four options based on frequency: score 0 (rarely or not at all, less than 1 day), score 1 (not too much, 1–2 days), score 2 (sometimes or about half the time, 3–4 days), and score 3 (most of the time, 5–7 days). The entries for reverse scoring are items 5 and 8. Total scores ranged from 0 to 30, with higher scores indicating poorer mental health. In particular, a score ≥ 10 was defined as the presence of depressive symptoms.

#### 2.2.4 Demographic characteristics

In this study, several key demographic characteristics were selected as control variables based on the relevant literature (25–27) and in context, including age, gender, marital status, drinking and smoking habits, exercise status, presence of chronic diseases, medical insurance and Pension insurance, education level, and life satisfaction.

### 2.3 Statistical analysis

Statistical analysis was performed using SPSS 27.0 software. Measurement information was expressed as mean and standard deviation; count information was expressed as frequency and percentage. The *t*-test or ANOVA was used to compare the differences in sleep duration among older adults with different characteristics of chronic diseases. Pearson’s correlation analysis was used to explore the correlation between pain and sleep, life satisfaction, and activity of daily living, and the process macros developed by Hayes et al. were used to calculate the 95% confidence level by using the bias-corrected non-parametric percentile bootstrap method (Model6, with repetitive sampling 5,000 times). The mediation effect test was performed using the bias-corrected non-parametric percentile bootstrap method (Model6, 5,000 replications, 95% confidence intervals calculated) with a test level of *α* = 0.05.

## 3 Results

### 3.1 Descriptive statistics on the basic characteristics of the rural older adults

A total of 5,352 study participants were included in this study, of whom 3,201 (59.81) were aged 60–69 years, 1,823 (34.06) were aged 70–79 years, and 328 (6.13) were aged 80 years and above, with 2,585 (48.3%) females and 2,767 (51.7%) males. The average length of sleep was 5.99 ± 2.07 h. Among the study participants, 81% were married and most of the participants had chronic diseases (85.6%). In terms of lifestyle, 65.7% (3,515) of the participants did not consume alcohol, 72.1% (3,860) did not smoke, while 87.6% (4,689) participated in regular exercise. In addition, 94.9% of the rural older adult had medical insurance and 87.4% had pension insurance. In terms of education level, the group with lower education is more prominent, with 56.6% (3,030) of the participants having elementary school education or below. Regarding life satisfaction, 51.0% (2,728 people) said they were relatively satisfied and 33.6% (1,799 people) said they were very satisfied (see Table 1).

**Table 1:** Differences in sleep duration among rural older adults with different demographic characteristics (*N* = 5,352).

Variables	Group	*N* (%)	Sleep duration (hours)	*t/F*	*P*
			$\bar{x}$ *± s*		
Age	60–69 = 1	3,201(59.8)	6.01 ± 1.94	1.158	0.314
	70–79 = 2	1,823(34.1)	5.97 ± 2.21		
	80 years and above = 3	328(6.1)	5.84 ± 2.41		
Gender	Female = 0	2,585(48.3)	5.66 ± 2.16	−11.298	<0.001
	Male = 1	2,767(51.7)	6.30 ± 1.93		
Marital status	Other = 0	1,015(19.0)	5.71 ± 2.27	−4.483	<0.001
	Married = 1	4,337(81.0)	6.02 ± 2.02		
Chronic diseases	No = 0	769(14.4)	6.39 ± 1.91	6.224	<0.001
	Yes = 1	4,583(85.6)	5.92 ± 2.09		
Drinking	No = 0	3,515(65.7)	5.94 ± 2.12	−2.367	0.018
	Yes = 1	1,837(34.3)	6.08 ± 1.97		
Smoking	No = 0	3,860(72.1)	5.87 ± 2.11	−6.810	<0.001
	Yes = 1	1,492(27.9)	6.29 ± 1.94		
Exercise status	No = 0	663(12.4)	5.94 ± 2.34	−5.535	0.593
	Yes = 1	4,689(87.6)	6.00 ± 2.03		
Medical insurance	No = 0	272(5.1)	6.10 ± 2.42	0.811	0.418
	Yes = 0	5,080(94.9)	5.98 ± 2.05		
Pension insurance	No = 0	672(12.6)	5.92 ± 2.05	−0.997	0.319
	Yes = 1	4,680(87.4)	6.00 ± 2.07		
Education level	Below primary school = 1	3,030(56.6)	5.85 ± 2.20	15.909	<0.001
	Primary school = 2	1,166(21.8)	6.07 ± 1.96		
	Middle school = 3	783(14.6)	6.22 ± 1.83		
	High school and above = 4	373(7.0)	6.39 ± 1.63		
Life satisfaction	Not at all satisfied = 1	162(3.0)	5.14 ± 2.29	14.840	<0.001
	Not very satisfied = 2	442(8.3)	5.49 ± 2.20		
	More satisfied = 3	2,728(51.0)	5.99 ± 1.98		
	Very satisfied = 4	1,799(33.6)	6.16 ± 2.07		
	Extremely satisfied = 5	221(4.1)	6.24 ± 2.35		

### 3.2 Between-group comparisons of sleep duration among rural older adults

The results of t-test or ANOVA comparison showed statistically significant differences (*p* < 0.05) in sleep duration among rural older adults across gender, marital status, presence of chronic diseases, smoking and drinking, education level, and life satisfaction, which were included as covariates in the regression model (see Table 1).

### 3.3 Correlation analysis

Among the 5,352 older adults, the number of pains was significantly negatively correlated with the number of hours of sleep (*r* = −0.212), and significantly positively correlated with the impairment to activity of daily living (*r* = 0.339) and depression (*r* = 0.355); there was a positive correlation between the impairment to activity of daily living and depression (*r* = 0.319) and a negative correlation with the number of hours of sleep (*r* = −0.159); depression was negatively correlated with the number of hours of sleep (*r* = −0.268), and all correlations were statistically significant (*p* < 0.01) (see Table 2).

**Table 2:** Results of correlation analysis.

Variables	1 Pain	2 Sleep duration	3 ADL	4 Depression
1. Pain	1.000			
2. Sleep duration	−0.212**	1.000		
3. ADL	0.339**	−0.159**	1.000	
4. Depression	0.355**	−0.268**	0.319**	1.000

**p < 0.01.

### 3.4 Analysis of intermediation effects

In this study, we analyzed the chain-mediated effects of activity of daily living and depression on pain and sleep duration among rural older adults, controlling for gender, marriage, presence of chronic diseases, alcohol and smoking status, education level, and life satisfaction, with pain as the independent variable and sleep duration as the dependent variable. The results showed that pain condition negatively predicted sleep duration (*β* = −0.060) and positively predicted activity of daily living (*β* = 0.141) and depression (*β* = 0.366); activity of daily living positively predicted depression (*β* = 0.694) and negatively predicted sleep duration (*β* = −0.063); and depression negatively predicted sleep duration (*β* = −0.059), and all the results were statistically significant (*p* < 0.05) (see Table 3).

**Table 3:** Results of mediation analysis.

Variables	Model 1 ADL			Model 2 depression			Model 3 Sleep duration		
	*β*	*SE*	*t*	*β*	*SE*	*t*	*β*	*SE*	*t*
Constant	1.099	0.128	8.591^***^	17.962	0.454	39.592^***^	6.449	0.182	35.51^***^
Pain	0.141	0.006	22.609^***^	0.366	0.023	15.904^***^	−0.060	0.008	−7.19^***^
ADL				0.694	0.048	14.411^***^	−0.063	0.017	−3.636^***^
Depression							−0.059	0.005	−12.189^***^
Gender	0.080	0.056	1.426	−1.323	0.198	−6.674^***^	0.388	0.070	5.539^***^
Marital status	−0.147	0.056	−2.624^**^	−1.224	0.198	−6.18^***^	0.117	0.070	1.669
Chronic diseases	0.253	0.064	3.984^***^	1.257	0.224	5.606^***^	−0.081	0.079	−1.023
Drinking	−0.103	0.050	−2.061^*^	−0.319	0.176	−1.812	−0.222	0.062	−3.592^***^
Smoking	−0.155	0.056	−2.769^**^	0.139	0.198	0.704	0.077	0.070	1.110
Education level	−0.063	0.025	−2.587^*^	−0.591	0.086	−6.835^***^	0.001	0.031	0.040
Life satisfaction	−0.216	0.028	−7.814^***^	−2.357	0.098	−24.113^***^	0.053	0.036	1.454
*R^2^*	0.133			0.283			0.100		
*F*	102.036^***^			234.115^***^			59.152^***^		

^*^P < 0.05, ^**^p < 0.01, ^***^p < 0.001.

### 3.5 Significance test for mediating effects

Significance tests were performed using the bootstrap method, which generates 5,000 random samples from the raw data. A mediating effect was considered significant if the 95% confidence interval (*CI*) derived from these samples did not include zero. The results of the mediated effects test showed that the direct effect of pain on sleep duration was statistically significant (95% *CI* = −0.076 to −0.043) and accounted for 62.50% of the total effect, while activity of daily living and depression had statistically significant mediated effects of pain and sleep duration (95% *CI* = −0.014 to −0.004; 95% *CI* = −0.026 to −0.017), accounting for 9.38 and 21.87% of the total effect, respectively; and the chained mediated effect of activity of daily living and depression was statistically significant for pain and sleep duration in rural older adults (95% *CI* = −0.007 to −0.004), accounting for 6.25% of the total effect (see Table 4).

**Table 4:** Significance tests for mediating effects.

Effect	Paths	*B*	*SE*	95%*CI*	Ratio (%)
Direct effect	Pain → Sleep duration	−0.060	0.008	(−0.076, −0.043)	62.50
Indirect effect	Pain → ADL → Sleep duration	−0.009	0.003	(−0.014, −0.004)	9.38
	Pain → Depression → Sleep duration	−0.021	0.002	(−0.026, −0.017)	21.87
	Pain → ADL → Depression → Sleep duration	−0.006	0.001	(−0.007, −0.004)	6.25
Total indirect effect		−0.036	0.004	(−0.043, −0.029)	37.50
Total effect	Pain → Sleep duration	−0.096	0.008	(−0.111, −0.080)	100.00

## 4 Discussion

### 4.1 Relationship between pain and sleep duration

The results of this study showed that there was a significant negative correlation between the number of pain sites and sleep duration, and the higher the number of pain sites, the lower the sleep duration. This finding is consistent with previous studies (28, 29), suggesting that Pain not only affects individuals physically but also disrupts sleep through central nervous system interactions. From the point of view of physiological mechanisms, there is an interaction between pain and sleep through the regulation of the central nervous system. It has been noted (30) that pain perception signals transmit peripheral noxious stimulus signals through the spinal cord to the brain, affecting neurotransmitter release and thus interfering with sleep. As the number of pain sites increases, the superimposed effect of pain signals may intensify the stimulation of the brain, and pain signals from different sites may trigger a wider range of neural activity, further exacerbating the negative impact on sleep and thus reducing sleep duration. In addition, studies have shown that pain often leads to sleep fragmentation in patients (31), prolongs the delay in falling asleep (32), and increases the frequency of nocturnal awakenings (33), resulting in their poorer sleep, which in turn affects the reduction of total sleep duration (34). Rocio de la Vega and others have also demonstrated that people with the presence of multiple sites of pain usually report poorer sleep quality and that this sleep disorder significantly affects their sleep duration (35).

### 4.2 Mediation effect analysis

The mediating effects analysis in this study further revealed the complex mechanisms underlying the effects of pain on sleep duration. First, the activity of daily living played a significant mediating role between pain and sleep. Pain is not only a common and persistent health problem among older adults, but also one of the major contributing factors to disability, often interfering with the activity of daily living (36). It has been shown that pain can significantly limit the ability of older adults to perform voluntary activities and may even lead to further deterioration of physical function, thus creating a vicious cycle that ultimately has a negative impact on sleep (37, 38). Second, depression as another mediating variable played a key role in the relationship between pain and sleep. The results of this study showed that pain not only had a significant negative impact on sleep, but also indirectly affected sleep through depression. It has been established that pain triggers unpleasant sensations and disturbing emotions that lead to feelings of helplessness and hopelessness in individuals, and these emotions can exacerbate the development of depressive symptoms (39, 40), which are risk factors for developing depression. Whereas depression can be more likely to lead to the development of sleep disorders, significantly affecting sleep duration (41, 42)^.^ In addition the present study further revealed the progressive roles of activity of daily living and depression in the relationship between pain and sleep duration through chain-mediated modeling. When suffering from pain, older adults’ self-care capacity limitations prevent them from completing daily activities (43), which in turn leads to emotions such as anxiety and depression (44, 45), which can further negatively impact sleep length. This chained pathway highlights the interaction between activity of daily living and depression, emphasizing the need to integrate physical and psychological dimensions when understanding the multidimensional impact of pain on health in older adults.

### 4.3 Implications

The results of the study have significant implications for the health of rural older adults, and in conjunction with the full paper, we can formulate recommendations to address the above issues, focusing on the following three areas. Firstly, the government and society should provide more precise policy support and resource investment (46, 47). They should establish and improve basic medical and health service facilities, increase the publicity of public health policies, raise the awareness of pain, depression, disability and sleep problems among rural older adult groups, and promote their active participation in health management and intervention. Second, village-level organizations or communities should improve the system of care services for the older adults in rural areas. They should establish and make effective use of activity rooms for the older adults, organize related cultural and recreational activities, and pay attention to the living and health conditions of rural older adults with impaired health. By providing relevant services and cultural and recreational activities, the loneliness of the older adults can be alleviated and their spiritual well-being can be enhanced (48). Finally, family old-age care is the main old-age care in rural areas, and family members (such as children) should be encouraged to provide more emotional support and maintain a positive and optimistic mindset (4).

### 4.4 Limitations

The sample size included in this study covers most of the provinces in China and is representative of the rural middle-aged and older population in China to a certain extent, but there are still some limitations. First, some of the data came from self-reporting by rural older adults, which may have subjective bias due to cultural background and other potential influencing factors that were not included. Second, no distinction was made between pain type and site, and the pain level was measured only by the number of pain sites, which may affect the precision of pain assessment, and the classification of the pain type can be refined or analyzed for the specific pain site in the future. Third, this study did not adequately consider the impact of potential confounding factors, such as socioeconomic factors, on health outcomes. Future studies should incorporate these factors into the analytic framework to improve the comprehensiveness and accuracy of the findings. Finally, this study used cross-sectional data, which could not reveal causal relationships. Follow-up studies could further explore causal mechanisms by tracking changes in individuals or groups at different points in time through longitudinal data.

## 5 Conclusion

In this study, we analyzed data from 5,352 older adults in the China Health and Aging Tracking Survey to explore the effect of pain on sleep duration among rural older adults, and further analyzed the chain-mediated roles of activity of daily living and depression in this relationship. The results of the study showed that there was a significant negative correlation between pain and sleep duration, and that activity of daily living and depression played chain-mediated roles in the relationship between pain and sleep duration.

Based on the findings, interventions to improve the health of rural older adults are urgent. Comprehensive intervention programs that focus on both pain management and improvement of daily mobility, mental health, and sleep quality should be implemented to reduce health risks and promote overall health. This provides a theoretical basis and practical guidance for future promotion of health interventions for older adults in rural areas.

## Data Availability

Publicly available datasets were analyzed in this study. This data can be found: https://charls.pku.edu.cn.
